# Experience of symptom control, anxiety and associating factors in a palliative care unit evaluated with Support Team Assessment Schedule Japanese version

**DOI:** 10.1038/s41598-021-97143-4

**Published:** 2021-09-29

**Authors:** Tetsuya Ito, Emi Tomizawa, Yuki Yano, Kiyozumi Takei, Naoko Takahashi, Fumio Shaku

**Affiliations:** 1 Department of Palliative Care, Japanese Red Cross Medical Center, 4-1-22 Hiroo, Shibuya-ku, Tokyo, 150-8935 Japan; 2 Department of Palliative Medicine and Advanced Clinical Oncology, IMSUT Hospital, The Institute of Medical Science, The University of Tokyo, Tokyo, Japan; 3 Department of Psychosomatic Internal Medicine, Nihon University Itabashi Hospital, Tokyo, Japan

**Keywords:** Disability, Oedema, Pain, Respiratory signs and symptoms, Cancer, Cancer, Psychology, Signs and symptoms

## Abstract

Various physical and psychosocial difficulties including anxiety affect cancer patients. Patient surroundings also have psychological effects on caregiving. Assessing the current status of palliative care intervention, specifically examining anxiety and its associated factors, is important to improve palliative care unit (PCU) patient quality of life (QOL). This study retrospectively assessed 199 patients admitted to a PCU during August 2018–June 2019. Data for symptom control, anxiety level, disease insight, and communication level obtained using Support Team Assessment Schedule Japanese version (STAS-J) were evaluated on admission and after 2 weeks. Palliative Prognostic Index (PPI) and laboratory data were collected at admission. Patient anxiety was significantly severer and more frequent in groups with severer functional impairment (*p* = 0.003) and those requiring symptom control (*p* = 0.006). Nevertheless, no relation was found between dyspnea and anxiety (*p* = 0.135). Patients with edema more frequently experienced anxiety (*p* = 0.068). Patient survival was significantly shorter when family anxiety was higher after 2 weeks (*p* = 0.021). Symptoms, edema, and disabilities in daily living correlate with patient anxiety. Dyspnea is associated with anxiety, but its emergence might be attributable mainly to physical factors in this population. Family members might sensitize changes reflecting worsened general conditions earlier than the patients.

## Introduction

Cancer patients experience various difficulties that adversely affect their quality of life (QOL)^1–4^. Anxiety reportedly exists more frequently among cancer patients^5^. Cancer survivors often require the use of medication^6^. Chen et al*.* reported that anxiety is negatively correlated with spiritual well-being^7^, and that it is associated with worse QOL in patients after chemotherapy^8^. These findings suggest anxiety as a severe difficulty for cancer patients and survivors.

Regarding physical symptoms, pain, a commonly occurring symptom among cancer patients, is reported by 55.0% of patients during cancer treatment and by 66.4% of patients in advanced or terminal stages^9^. Dyspnea, known as a prognostic predictive factor^10^, has prevalence correlated with disease progression^11^. Fatigue is also a frequently observed symptom in cancer patients^12^, but no treatment strategy has yet been established for it^13^. These symptoms, including pain, dyspnea, and fatigue, reportedly interfere with patients’ daily living^14^.

Physical symptoms that might coexist with psychosocial difficulties, not limited to anxiety, are also known to occur frequently in palliative care settings. Hui et al. reported that 44% of patients admitted to an acute PCU experience spiritual distress; they also reported its relation to pain^15^. Correlation of psychological stress with dyspnea has also been reported^16^. These facts suggest that psychological problems and physical symptoms are closely related and that consideration must include both perspectives.

Patients with life-threatening disease such as cancer might have also difficulties with the recognition process of prognosis. Better understanding of the process can be expected to help medical professionals provide personalized care and support decision-making^17^. This finding indicates that it is important to examine patients’ insight into diseases, which might reflect psychological acceptance of diseases.

Relationships between patients and their surrounding people are reportedly important for spiritual care^18^. Co-existence should be specifically addressed in caring for patients with anxiety^19^. Communication and relationships with healthcare professionals are also fundamentally important for patients’ decision making^20^. According to these findings, it is also important to examine their social status, including their relationships with surrounding people, when considering the quality of palliative care, including psychological aspects.

Along with the patients themselves, their caregivers, including family members, are also facing difficulties that include psychological distress^21–25^. Cancer patient family caregivers also have anxiety^21^. Amelioration of their psychological difficulties might raise care quality^26^. Bereavement support for caregivers is also known to reduce their grief, depression, and anxiety^27^. Early recognition of their difficulties, including anxiety, might also help establish family psychological care. In palliative care settings, particularly addressing the caregiver's anxiety is important because it results not only in better patient care, but also helps them cope with their psychological difficulties during patient care, and even after a patient's death.

As described above, cancer patients are affected by difficulties including psychological and physical factors that might be coexisting or cross-interacting. Their surrounding people are also affected during caregiving. Palliative care services are intended to improve psychosocial difficulties as necessary in clinical settings and also to improve the QOL of the patients and their families through control of physical difficulties including pain and other symptoms^4,28^. Comprehensive understanding of palliative care status is necessary to support patients and their families effectively. This study was conducted to assess the current status of palliative care intervention, with specifically examine anxiety and its associated factors.

## Methods

This retrospective observational study was conducted at a single center. Medical records of patients admitted to the PCU at Japanese Red Cross Medical Center during August 2018–June 2019 were surveyed. Patients with multiple admissions were recruited only at their first admission during the observational period. Patient survival was observed until March 2020.

On admission, patient background data including age, sex, primary cancer site, length of hospital stay, and disease duration were collected. Data for the Palliative Prognostic Index (PPI)^10^, laboratory data including white blood cell count, lymphocyte percentage, C-reactive protein (CRP) concentration, and the Support Team Assessment Schedule Japanese version (STAS-J)^29^ were also collected.

Actually, PPI is a reliable and simple tool to assess the life-expectancy of cancer patients^10^. Calculated according to performance status evaluated with the palliative performance scale (PPS)^30^, oral intake, dyspnea at rest, delirium, and edema, a total score of more than 6 or 4 of the maximum 15 indicates survival of 3 or 6 weeks. The cut off was set as described in an earlier report^10^.

Elevated white blood cell counts and low lymphocyte percentage are known as factors predictive for poor prognosis^31^. They indicate inflammation under certain conditions, which is also reportedly associated with anxiety^32–34^. Association between CRP and anxiety has been described earlier in the literature^35,36^. Therefore, CRP was selected in addition as a marker of general inflammation, also in association with anxiety.

A reliable surrogate evaluation tool, STAS, is useful to assess the existence of difficulties and the need for their improvement in palliative settings^37^. Since it was first developed, the tool has been used widely in Japan and other countries^29,38,39^. Of 16 items evaluated using the original STAS, core items including control of pain and other symptoms, anxiety, insight of prognosis, and communication level of the patients, their family members and professionals are evaluated with STAS-J. From these points of view, STAS-J is suitable. It was selected for this survey. Another seven items including planning, practical aid, financial, wasted time, spiritual, professional anxiety and advising professionals are not evaluated with STAS-J, which might address the physical and psychological difficulties of patients and their families more directly. The status of each difficulty and its need for improvement is evaluated with a five-point rank scale (0 through 4, more difficulties denoted by a higher score) by medical staff in charge. For this study, evaluation with STAS-J was performed always by several, at least three, palliative care staff members including palliative physicians and nursing staff members. Although STAS is not a subjective patient-reported outcome scale, its usefulness should be emphasized when considering the condition of patients in palliative care settings who might be delirious or in a drowsy state. Regarding the observation period required for symptom control, although effects of opioids against dyspnea can be evaluated within some hours to 2 days^40,41^, 14 days are necessary for opioid rotation against cancer pain treatment^42^. A 2-week period is also applied in some studies evaluating transitional changes of anxiety^43,44^. Considering also the mean and median hospital stay of the participants in addition, data for STAS-J after 2 weeks of admission were collected again to assess their transitional change. Considering that the accuracy of the statistics could not be ensured because of the paucity of cases, we did not conduct our evaluation over a longer period of time.

Palliative care services including symptom control and psychosocial intervention were administered by multidisciplinary staff members including palliative physicians, nursing staff, dental hygienists, psychotherapists, music therapists, and a harp therapist.

To compare the change of STAS-J score from that at admission to that after 2 weeks, the Wilcoxon signed rank test, a paired nonparametric test, was used because these scores are paired categorical rank data. A two-group comparison of STAS-J scores related to the severity and frequency of anxiety in patients who require some symptom control (STAS-J: pain or other symptom control ≥ 2) versus those in other patients was performed using the Wilcoxon rank sum test, an unpaired nonparametric test, because these scores are unpaired categorical rank data. The Wilcoxon rank sum test was also used for two-group comparison of items including other unpaired categorical rank data for which normality cannot be assumed, such as the PPI total score, edema, dyspnea at rest, delirium, and laboratory data. When the factor was classified into two categories such as communication level, insight of disease and anxiety, a two-group comparison was applied using Fisher’s exact test. That test is an accurate method for 2 × 2 contingency tables. For a three-group comparison of items such as PPS scores and oral intake, the Kruskal–Wallis test, a nonparametric multigroup test, was used. The log-rank test, which is commonly used in survival time analysis, was used to compare the survival distributions of patients between two groups such as symptom control or control required, and with anxiety or without anxiety. A *P* value of less than 0.05 was inferred as significant. Statistical analyses were conducted independently by the Japan Institute of Statistical Technology (Tokyo, Japan) using software (SPSS Statistics 23; IBM Corp., Armonk, NY, USA).

The entire protocol for this study was approved by the ethics committee of the Japanese Red Cross Medical Center (approval number 1006). This study was conducted in accordance with standards of the Declaration of Helsinki. In a non-invasive observational study, Japanese law requires no written informed consent from individual participants. Therefore, as approved by the ethical committee of the Japanese Red Cross Medical Center, an opt-out method was applied for this study rather than obtaining written informed consent.

## Results

### Patient characteristics

During the study period, 199 patients were admitted to the PCU of Japanese Red Cross Medical Center. As presented in Table 1A,B, they were 87 men and 112 women with mean age of 70.9 ± 12.7 years. The most common primary site was the lung (40 of 199 patients).

**Table 1:** Patient characteristics.

(A) Patient background	*n*	Mean ± SD (median; range) or number (%)
Age (y.o.)	199	70.9 ± 12.7 (72; 18–97)
Sex	199	Male, 87 (43.7)/ Female, 112 (56.3)
Primary site	199	Lung, 40 (20.1) / Pancreas, 32 (16.1) / Colorectal, 20 (10.5) / Gastric (including 1 GIST), 19 (9.5) / Hepatobiliary, 18 (9.0) / Breast, 15 (7.5) / Hematological, 10 (5.0) / Oral and Maxillofacial, 9 (4.5) / Esophagus, 7 (3.5) / Uterine, 5 (2.5) / Ovary, 4 (2.0) / Thyroid, 3 (1.5) / Prostate, 3 (1.5) / Cerebral, 2 (1.0) / Thymus, 2 (1.0) / Cecum, 2 (1.0) / Pleural mesothelioma, 1 (0.5) / Renal, 1 (0.5) / Renal pelvis, 1 (0.5) / Testicular, 1 (0.5) / Others, 3 (1.5) / Unknown, 1 (0.5)

(B) Hospital stay, disease duration and inflammatory markers		
	*n*	Mean ± SD (median; range)
Hospital stay (days)	199	22.3 ± 21.8 (15; 0–147)
Disease duration (months)	128	27.0 ± 30.1 (18; 1–236)
Laboratory test		
WBC (× 10^3^/μL)	148	12.4 ± 24.7 (8.9; 1.6–297.9)
Ly (%)	128	10.8 ± 9.8 (7.6; 0.5–59.3)
CRP (mg/dL)	145	6.9 ± 6.8 (4.7; 0.0–32.5)

(C) PPI score on admission	*n*	Mean ± SD (median; range) or score: *n* (%)
PPI Score Total	189	6.1 ± 3.0 (6.0; 0–12.5)
Items for PPI		
PPS	197	0: 17 (8.6) / 2.5: 136 (69.0) / 4.0: 44 (22.3)
Oral intake	198	0: 31 (15.7) / 1.0, 84 (42.4) / 2.5: 83 (41.9)
Edema	193	0: 88 (45.6) / 1.0: 105 (54.4)
Dyspnea	196	0: 161 (82.1) / 3.5: 35 (17.9)
Delirium	194	0: 153 (78.9) / 4.0: 41 (21.1)

Mean hospital stay and disease durations were, respectively, 22.3 ± 21.8 days (*n* = 199) and 27.0 ± 30.1 months (*n* = 128). Of 199 participants, 165 died during the first stay at our facility, 4 transferred to other hospitals, and 30 returned to their homes and nursing facilities. Of 34 patients who survived to discharge, 26 were found to have died after discharge during the follow up period. Laboratory tests on admission yielded the following results: white blood cell count of 12.4 ± 24.7 × 10^3^/μL (*n* = 148), lymphocyte percentage of 10.8 ± 9.8% (*n* = 128), and CRP concentration of 6.9 ± 6.8 mg/dL (*n* = 145). The total PPI score was 6.1 ± 3.0 (*n* = 189) on admission, as shown in Table 1C.

### STAS-J score on admission and after 2 weeks

Pain and other symptoms requiring control (STAS-J: pain and other symptom control ≥ 2) were reported by 29.8% (50 of 168) and 57.4% (93 of 162) of patients on admission. Increased anxiety (STAS-J: anxiety ≥ 2) was reported by 26.9% (32 of 119) of patients, and by 26.3% (30 of 114) of families. In addition, eight patients were experiencing higher anxiety (STAS-J: anxiety ≥ 2), as reported by both patients and family members. Difficulties in disease insight ranked as 1 and over were reported by 29.2% (26 of 89) of patients and by 10.6% (11 of 104) of family members. Regarding the communication level, no difficulty was ranked as 0 in 76.7% (92 of 120) between patients and family members, and in 90.7% (98 of 108) between patients and families and staff. Communication difficulties between medical staff members were not sensitized by 94.0% (158 of 168).

After 2 weeks, pain and other symptoms requiring control were reported by 24.1% (14 of 58) and 48.3% (28 of 58) of patients. Increased anxiety was reported by 28.9% (13 of 45) of patients and by 21.2% (11 of 52) of families. Difficulty in disease insight was reported by 30.0% (12 of 40) of patients and by 12.2% (6 of 49) of families. Regarding the communication level, no difficulty was found in 81.6% (40 of 49) between patients and family members, in 88.9% (48 of 54) between patients and families and staff, and in 96.5% (55 of 57) between medical staff members. No significant difference was found in the distributions of STAS-J scores between those on admission and those found after 2 weeks: pain, *p* = 0.400; other symptoms, *p* = 0.052; patient anxiety, *p* = 0.462; family anxiety, *p* = 0.499; patient insight of disease, *p* = 0.726; family insight of disease, *p* = 0.317; communication between patient and family, *p* = 1.000; communication between professionals, *p* = 0.527; communication of professional to patient and family, *p* = 0.453; Wilcoxon signed rank test.

### Anxiety and other factors evaluated using STAS-J

Relations between patient anxiety and symptom control evaluated with STAS-J are presented in Table 2. For patients requiring control of any symptom (STAS-J: pain or other symptom control ≥ 2), the severity and frequency of anxiety was significantly higher than for others (*p* = 0.006*, Wilcoxon rank sum test). No significant relation was found between symptom control and anxiety of their family members (*p* = 0.459. Wilcoxon rank sum test). Regarding communication between patients and their family members, no significant relation to anxiety of patients and their family members was found (*p* = 0.406 and 0.427, respectively; Fisher’s exact test). No significant relation was found between patient insight of disease and anxiety (patients, *p* = 1.000; family, *p* = 0.349; Fisher’s exact test), or family insight of disease and anxiety (patients, *p* = 0.741; family, *p* = 1.000; Fisher’s exact test).

**Table 2:** Patient anxiety on admission by symptom control.

Pain and Other symptoms		Patient anxiety on admission									Total
		0		1		2		3		4	
Both < 2 Symptom under control	*n* (%)	4 (11.8%)		26 (76.5%)		3 (8.8%)		1 (2.9%)		0 (0.0%)	34 (100.0%)
Others Symptom control required	*n* (%)	3 (3.6%)		53 (63.1%)		17 (20.2%)		9 (10.7%)		2 (2.4%)	84 (100.0%)
Total	*n* (%)	7 (5.9%)		79 (66.9%)		20 (16.9%)		10 (8.5%)		2 (1.7%)	118 (100.0%)

*p* = 0.006* (Wilcoxon rank sum test).

### Anxiety and laboratory data including inflammation markers

Table 3 presents a relation between patient anxiety evaluated with STAS-J and laboratory data including inflammation markers. Between patients with and without higher anxiety (STAS-J: patient anxiety ≥ 2 and others), no significant difference was found for WBC, Ly, or CRP (*p* = 0.660, 0.445, 0.507, respectively; Wilcoxon rank sum test).

**Table 3:** Inflammatory markers and patient anxiety evaluated with STAS-J on Admission.

Laboratory test	Patient anxiety ≥ 2			Patient anxiety < 2			Wilcoxon rank sum test (*p*)
	Mean ± SD	(Median; range)	*n*	Mean ± SD	(Median; range)	*n*	
WBC (× 103/μL)	10.0 ± 5.5	(8.2; 3.1–24.8)	26	9.5 ± 6.2	(8.3; 2.7–42.9)	68	0.660
Ly (%)	10.4 ± 9.4	(8.3; 2.0–36.0)	22	11.0 ± 8.3	(7.9; 1.0–50.7)	62	0.445
CRP (mg/dL)	6.1 ± 6.5	(4.5; 0.4–26.9)	26	6.7 ± 6.5	(4.6; 0.0–32.5)	68	0.507

### Anxiety and PPI score

Relations between PPI scores and anxiety of patients evaluated with STAS-J are shown in Table 4A–G. No significant relation was found between the PPI total score and anxiety of patients when the cut-off was set as > 6 or > 4: a cutoff of more than 6 showed *p* = 0.630; a cutoff of more than 4 was found with *p* = 0.429, by Wilcoxon rank sum test. Considering each item of PPI, the severity and frequency of patient anxiety was higher in cases with higher patient PPS scores reflecting a loss of daily living capabilities (*p* = 0.003*, Kruskal–Wallis test). In addition, anxiety tended to be experienced more severely and more frequently in patients with edema, but not to a significant degree (*p* = 0.068, Wilcoxon rank sum test). No significant relation between dyspnea at rest and anxiety of patients was found (*p* = 0.135, Wilcoxon rank sum test). Moreover, no significant relation was found between scores for oral intake, delirium, or anxiety: oral intake, *p* = 0.274, Kruskal–Wallis test; delirium, *p* = 0.559, Wilcoxon rank sum test. For family anxiety, there were no relating items in all and each score of PPI: total score cutoff of more than 6, *p* = 0.491, cutoff of more than 4, *p* = 0.581, Wilcoxon rank sum test; PPS, *p* = 0.181; oral intake, *p* = 0.788, Kruskal–Wallis test; edema, *p* = 0.473; dyspnea at rest, *p* = 0.444; delirium, *p* = 0.588; Wilcoxon rank sum test.

**Table 4:** Anxiety of patients evaluated with STAS-J and PPI.

**Patient Anxiety**		0	1	2	3	4	Total
**(A) Total score (cut off as 6)**							
PPI total score	≤ 6.0 *n* (%)	7 (8.9%)	50 (63.3%)	15 (19%)	6 (7.6)	1 (1.3%)	79 (100.0%)
	> 6.0 *n* (%)	1 (2.9%)	24 (70.6%)	4 (11.8%)	4 (11.8%)	1 (2.9%)	34 (100.0%)
Total	*n* (%)	8 (7.1%)	74 (65.5%)	19 (16.8%)	10 (8.8%)	2 (1.8%)	113 (100.0%)
**(B) Total score (Cut off as 4)**							
PPI total score	≤ 4.0 *n* (%)	5 (11.9%)	26 (61.9%)	7 (16.7%)	3 (7.1%)	1 (2.4%)	42 (100.0%)
	> 4.0 *n* (%)	3 (4.2%)	48 (67.6%)	12 (16.9%)	7 (9.9%)	1 (1.4%)	71 (100.0%)
Total	*n* (%)	8 (7.1%)	74 (65.5%)	19 (16.8%)	10 (8.8%)	2 (1.8%)	113 (100.0%)
**(C) Score for PPS**							
PPI-PPS	0 *n* (%)	4 (33.3%)	7 (58.3%)	1 (8.3%)	0 (0.0%)	0 (0.0%)	12 (100.0%)
	2.5 *n* (%)	3 (3.3%)	59 (64.8%)	18 (19.8%)	9 (9.9%)	2 (2.2%)	91 (100.0%)
	4.0 *n* (%)	1 (6.7%)	13 (86.7%)	0 (0.0%)	1 (6.7%)	0 (0.0%)	15 (100.0%)
Total	*n* (%)	8 (6.8%)	79 (66.9%)	19 (16.1%)	10 (8.5%)	2 (1.7%)	118 (100.0%)
**(D) Score for oral intake**							
PPI-Oral intake	0 *n* (%)	3 (11.5%)	18 (69.2%)	3 (11.5%)	2 (7.7%)	0 (0.0%)	26 (100.0%)
	1.0 *n* (%)	4 (6.9%)	34 (58.6%)	13 (22.4%)	5 (8.6%)	2 (3.4%)	58 (100.0%)
	2.5 *n* (%)	1 (2.9%)	27 (77.1%)	4 (11.4%)	3 (8.6%)	0 (0.0%)	35 (100.0%)
Total	*n* (%)	8 (6.7%)	79 (66.4%)	20 (16.8%)	10 (8.4%)	2 (1.7%)	119 (100.0%)
**(E) Score for edema**							
PPI-Edema	0 *n* (%)	5 (9.3%)	38 (70.4%)	9 (16.7%)	1 (1.9%)	1 (1.9%)	54 (100.0%)
	1.0 *n* (%)	3 (4.9%)	38 (62.3%)	10 (16.4%)	9 (14.8%)	1 (1.6%)	61 (100.0%)
Total	*n* (%)	8 (7.0%)	76 (66.1%)	19 (16.5%)	10 (8.7%)	2 (1.7%)	115 (100.0%)
**(F) Score for dyspnea at rest**							
PPI-Dyspnea	0 *n* (%)	8 (8.3%)	64 (66.7%)	16 (16.7%)	7 (7.3%)	1 (1.0%)	96 (100.0%)
	3.5 *n* (%)	0 (0.0%)	15 (65.2%)	4 (17.4%)	3 (13.0%)	1 (4.3%)	23 (100.0%)
Total	*n* (%)	8 (6.7%)	79 (66.4%)	20 (16.8%)	10 (8.4%)	2 (1.7%)	119 (100.0%)
**(G) Score for delirium**							
PPI-Delirium	0 *n* (%)	7 (6.5%)	70 (65.4%)	19 (17.8%)	9 (8.4%)	2 (1.9%)	107 (100.0%)
	4.0 *n* (%)	1 (10.0%)	7 (70.0%)	1 (10.0%)	1 (10.0%)	0 (0.0%)	10 (100.0%)
Total	*n* (%)	8 (6.8%)	77 (65.8%)	20 (17.1%)	10 (8.5%)	2 (1.7%)	117 (100.0%)

(A) *p* = 0.630 (Wilcoxon rank sum test).

(B) *p* = 0.429 (Wilcoxon rank sum test).

(C) *p* = 0.003* (Kruskal–Wallis test).

(D) *p* = 0.274 (Kruskal–Wallis test).

(E) *p* = 0.068 (Wilcoxon rank sum test).

(F) *p* = 0.135 (Wilcoxon rank sum test).

(G) *p* = 0.559 (Wilcoxon rank sum test).

### Patient survival and anxiety

**Figure 1:**
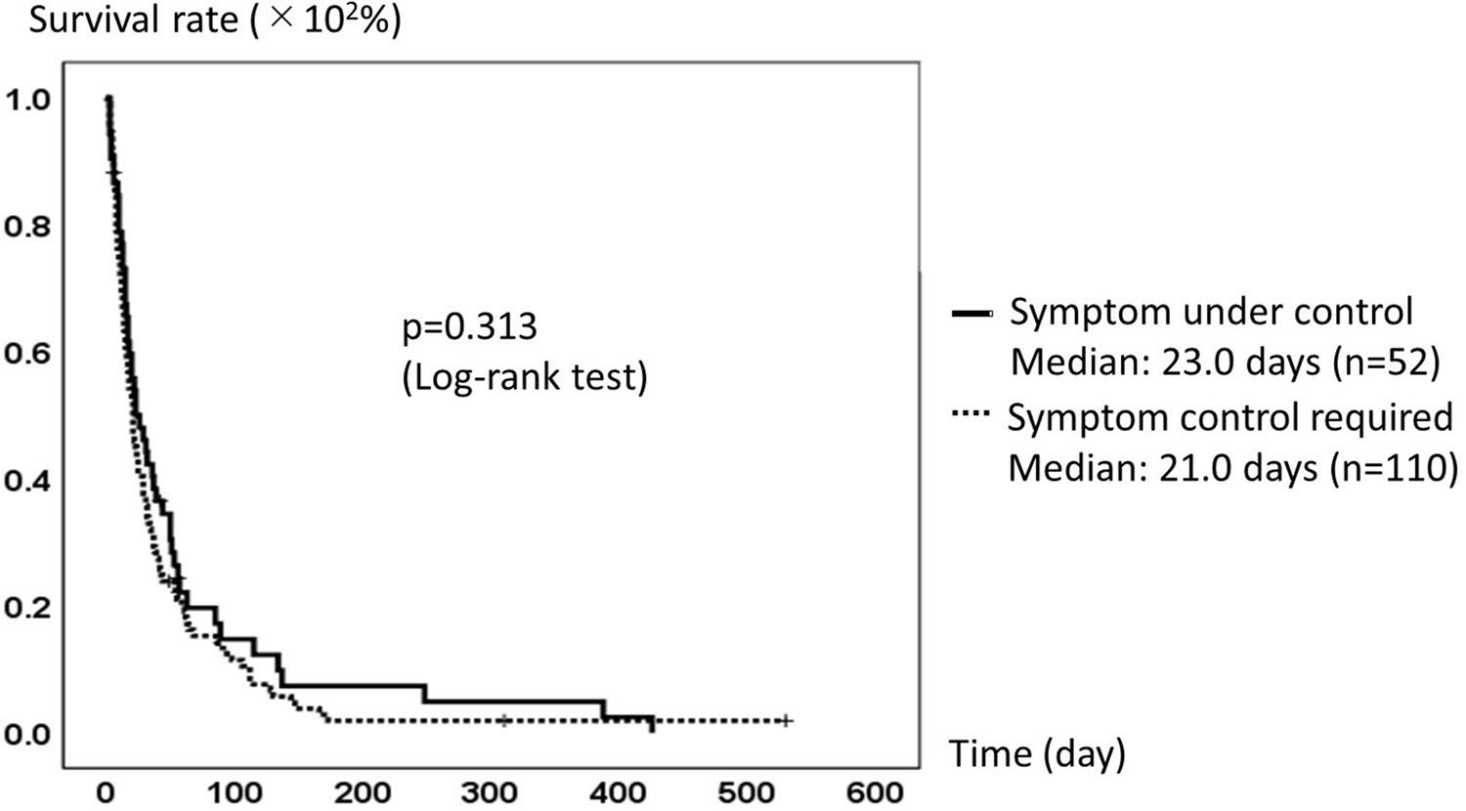
Patient survival and symptom control on admission. No significant relation was found between patient survival and the existence of any symptom requiring control defined by STAS-J: pain or other symptom control ≥ 2 with symptoms, median 21.0 days, *n* = 110 and without symptoms, median 23.0 days, *n* = 52, *p* = 0.313).

**Figure 2:**
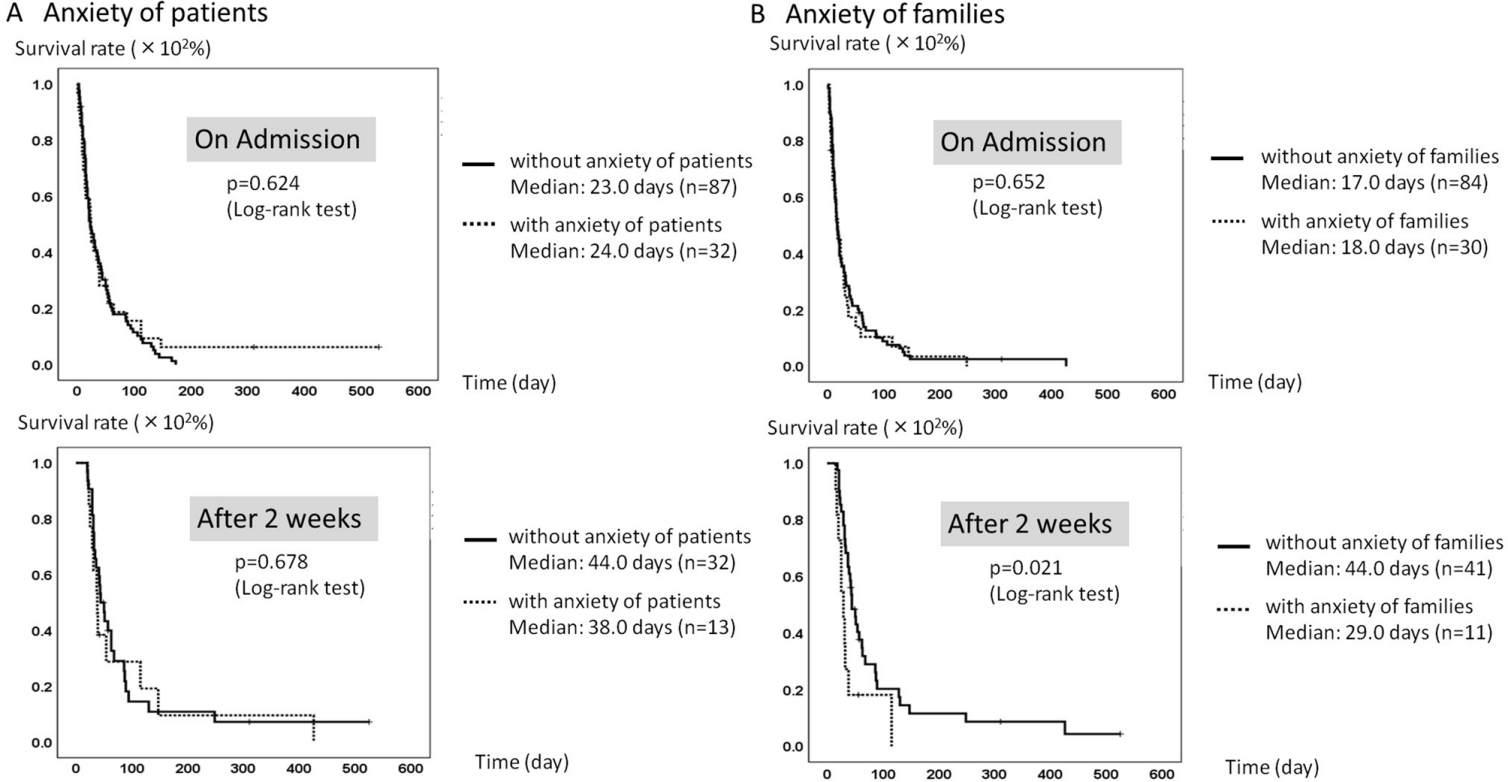
Patient survival and anxiety. (**A**) Anxiety of patients. No significant relation was found between patient survival and anxiety of patients on admission and after 2 weeks of admission: *p* = 0.624 and 0.678 respectively. Higher anxiety is defined as STAS-J: anxiety ≥ 2. (**B**) Anxiety of families. On admission, no significant difference was found in survival time between groups with and without higher anxiety of families defined as STAS-J anxiety ≥ 2: with, median 18.0 days, *n* = 30; without, median 17.0 days, *n* = 84; *p* = 0.652. However, significantly shorter survival was found when anxiety of family members was higher after 2 weeks: with higher anxiety, median 29.0 days, *n* = 11; without, median 44.0 days, *n* = 41; *p* = 0.021.

Patient survival and symptom control on admission are shown in Fig. 1. No significant difference was found for patient survival between groups with any symptom requiring control, as defined by STAS-J for pain or other symptom control ≥ 2 or without: with, median 21.0 days, *n* = 110; without, median 23.0 days, *n* = 52; *p* = 0.313, Log-rank test. Figure 2A,B present relations between patient survival and anxiety. Defining higher anxiety as STAS-J anxiety ≥ 2, no significant difference was found between patient survival and anxiety of patients on admission: with, median 24.0 days, *n* = 32; without, median 23.0 days, *n* = 87; *p* = 0.624, Log-rank test. Setting 2 weeks after as a starting point, no significant difference in survival time was found: with, median 38.0 days, *n* = 13; without, median 44.0 days, *n* = 32; *p* = 0.678, Log-rank test. Regarding anxiety of families, no significant difference was found in patient survival between groups with and without anxiety on admission: with, median 18.0 days, *n* = 30; without, median 17.0 days, *n* = 84; *p* = 0.652, Log-rank test. However, patient survival was significantly shorter in the group with higher anxiety of family members than without after 2 weeks: with, median 29.0 days, *n* = 11; without, median 44.0 days, *n* = 41; *p* = 0.021, Log-rank test.

## Discussion

This study has produced novel findings for difficulties experienced by patients admitted to a PCU and about the present status of palliative care.

### Transitional change of each STAS-J score during the first 2 weeks

Comparison of STAS-J scores on admission and after 2 weeks revealed no significant difference, which means that various symptoms, anxiety, insight into disease, and communication level neither improved nor worsened significantly during the first 2 weeks of stay at a PCU. Seow et al. reported rapid changes in ADL and symptoms of cancer patients at terminal phase^45^. Results of this study might reflect the emergence of such a phenomenon. Although no significant improvement was found, the fact that it did not worsen might be of some importance. It is also possible that the patient had already received possible interventions as required. This study found no communication problems in many cases on admission. Open communication and relationship with healthcare professionals are necessary for patients’ decision making^20^. In such processes, precise disease insight might be gained. For these patients and their families who have decided to be admitted to a PCU, communication between patients and their surroundings and disease insight might have already been established before admission. Although the results also indicate that greater effort is required, patient QOL seems to have been controlled overall in such situations. However, different results might be obtained with a longer observation period.

### Anxiety evaluated with STAS-J and associated factors

During the last months of life, rapid changes in ADL and physical symptoms emerge^45^. A higher total PPI score indicates shorter survival^10^. Elevated white blood cell counts and low lymphocyte percentages are also known to be predictive of poor prognosis^31^. They might indicate poor general condition, with poor prognosis. Comparing anxiety with these including PPI and laboratory data might indicate factors associated with anxiety of PCU patients. The relation between anxiety and inflammation has also been reported^32–34^, as have relations among inflammatory markers, association between CRP and anxiety in population-based studies^35,36^. White blood cell counts and lymphocyte percentages also indicate inflammation in addition to shorter survival. However, based on data obtained from this study, no significant difference was found in the total PPI score and laboratory data including CRP between groups with or without higher anxiety. These results suggest a lack of a clear contribution of the general condition and inflammation to the anxiety of patients and family members in this population. Participants of this study are facing life-threatening illness. Its emergence might be influenced by multiple factors. Moreover, it should be stated that these chosen markers are not necessarily specific markers for anxiety.

Comparison of anxiety levels with the respective PPI items shows that patients with higher PPS scores, indicating severer functional impairment, were found to feel anxiety more severely and frequently. Patients with edema also tended to feel anxious. Higher PPS scores reflecting loss of daily living capabilities are expected to contribute to the emergence of anxiety. Existence of edema might also be associated with the progress of anxiety affecting changes of their body images, causing heaviness and disability in daily living as well. This result is consistent with those obtained from an earlier study investigating the association of functional impairment with anxiety^46^. No relation was found between these two factors and the anxiety of their family members.

Hopwood et al*.* reported that burdens of physical symptoms predict depression in patients with lung cancer^47^. Psychological factors are also reportedly predictive for pain in cancer patients^48^. As described in these reports, relations between physical symptoms and psychosocial problems are known to exist. In this study, the frequency and severity of patient anxiety was found to be higher in patients with any symptom requiring control on admission. Nevertheless, these results do not show either as a cause or effect. The possibility of a close interrelation must be considered.

### Anxiety, communication environment and disease insight

Relations between patients and their family members and surrounding people reportedly play a key role in spiritual care^18^. Proper communication environments can mitigate spiritual pain and the existential suffering of patients. Nevertheless, no significant relation was found between the degree of familial communication and anxiety of patients. The importance of communication between cancer patients and physicians involving their families has also been reported^49^. Such communication might play important roles in caregiving. However, cases associated with difficulties were too few. Disease insight, which might reflect psychological acceptance of disease, was also unrelated to patient anxiety. For these patients and families to have decided on admission to a PCU, communication between patients and family members and disease insight might have already been established before admission, eliminating their association with anxiety. In this population setting, physical factors including irritability symptoms might play more important roles in the emergence of anxiety.

### Anxiety and symptom control, especially in relation to dyspnea

As described above, our data indicate a significant relation among symptoms requiring control and patient anxiety. Of the symptoms reported frequently by cancer patients, dyspnea is widely known to be associated with psychological problems including anxiety^16,50,51^, in addition to being a prognostic predictive factor^10^. Patients are probably sensitive to dyspnea as affected by various factors, including anxiety. In contrast, respiratory distress might engender anxiety, given that breathing is fundamentally important to sustain life. However, no association of the PPI-dyspnea at rest score was found with anxiety. Although dyspnea is evaluated using PPI only at rest, this result might indicate the greater importance of physical factors than anxiety in relation to dyspnea in this population setting.

### Emergence of anxiety in patients admitted to a PCU

As described above, it has been suggested that the involvement of ADL decline indicated by PPI and physical symptoms be regarded as factors causing anxiety in patients admitted to a PCU. Existence of edema might also contribute to patient anxiety, affecting ADL. In terms of reducing the anxiety of patients, this result suggests that efforts to improve ADL, such as rehabilitation or appropriate care, are necessary in addition to symptom control. No clear involvement of disease insight and communication environment was found from this study. As described above, their established status might account for this result.

### Patient survival and associated factors

No significant difference in patient survival was found between groups with and without higher anxiety of patients and family members on admission. However, at 2 weeks after admission, patient survival was significantly shorter when family members were feeling anxiety at a higher level and more frequently, but not the patients themselves. These results suggest that family members sensitize changes that reflect worsening of general conditions earlier than the patients themselves. That sensitization might also engender the prevalence of anticipatory grief of caregivers. This result indicates that more subtle psychological conflicts might arise in families. Moreover, it underscores the importance of devoting attention to this issue. These results also suggest the importance of specific examination of a patient’s anxiety irrespective of their expected survival to improve their QOL.

No significant difference was found between patient survival and the existence of any symptom requiring symptom control on admission, indicating the importance of symptom control irrespective of the expected survival.

### Limitations of this study

This study has some limitations. First, this is a retrospective observational study conducted at a single center, which means that the patient background is neither uniform nor generalized. Second, the patient-reported outcome scale was not applied in this study because it might be difficult to perform as a result of the poor general condition of the participants. This situation can also engender more missing data, especially when a patient is delirious or with somnolence. Moreover, it can be a factor leading to bias in evaluating physical and psychosocial difficulties. Third, longer-term observational evaluation with ensured statistical efficacy was not possible because of the reduced numbers of participants thereafter.

### Future perspective of this study, QOL of patients and families

The main target of palliative care is the QOL of the patient and family. Although QOL is an individual’s perception and STAS is not a tool for its assessment, problems evaluated with STAS include physical and psychological problems: the components of QOL^52^. The current states of difficulties experienced by patients and their families were presented in this report, along with their transitional changes. For some difficulties, related items were inferred. In addition, this report is the first of a study using STAS and indicating earlier family perception of worsening general conditions of patients at PCU.

To develop palliative care services, factors influencing care must be identified in future studies. Further prospective investigations based on patient-reported outcomes must be undertaken to improve the QOL of patients and their family members.

## Conclusion

Various symptoms and anxiety of cancer patients did not worsen during the first 2 weeks of stay at the PCU. Disability in daily living and the existence of symptoms might play a more important role in exacerbating psychological difficulties than the worsened general condition, inflammation, communication with surrounding people, or psychological acceptance of disease. Dyspnea is known to coexist with psychological distress. However, respiratory distress might also be caused mainly by physical factors in the population setting examined in this study. The results underscore the need to devote sufficient attention to physical symptoms and ability in daily living to improve patient comfort irrespective of the expected patient survival.

Family members of patients might sensitize any change reflecting general conditions earlier than the patients themselves. Sufficient attention must be devoted to patients’ families in psychological aspects to support caregivers and patients comprehensively.
